# Enhanced Thermal Conductivity of Epoxy Composites Filled with Al_2_O_3_/Boron Nitride Hybrids for Underfill Encapsulation Materials

**DOI:** 10.3390/polym13010147

**Published:** 2021-01-01

**Authors:** William Anderson Lee Sanchez, Chen-Yang Huang, Jian-Xun Chen, Yu-Chian Soong, Ying-Nan Chan, Kuo-Chan Chiou, Tzong-Ming Lee, Chih-Chia Cheng, Chih-Wei Chiu

**Affiliations:** 1 Department of Materials Science and Engineering, National Taiwan University of Science and Technology, Taipei 10607, Taiwan; williaxom@gmail.com (W.A.L.S.); d10504015@gapps.ntust.edu.tw (C.-Y.H.); ch60210@gmail.com (J.-X.C.); yuchiansoong@gmail.com (Y.-C.S.); 2 Material and Chemical Research Laboratories, Industrial Technology Research Institute, Hsinchu 31040, Taiwan; jan.in.nan@gmail.com (Y.-N.C.); JeffreyChiou@itri.org.tw (K.-C.C.); tzmlee@itri.org.tw (T.-M.L.); 3 Graduate Institute of Applied Science and Technology, National Taiwan University of Science and Technology, Taipei 10607, Taiwan; cccheng@mail.ntust.edu.tw

**Keywords:** underfill encapsulation, aluminum oxide, boron nitride, hybrid filler, thermal conductivity, coefficient of thermal expansion

## Abstract

In this study, a thermal conductivity of 0.22 W·m^−1^·K^−1^ was obtained for pristine epoxy (EP), and the impact of a hybrid filler composed of two-dimensional (2D) flake-like boron nitride (BN) and zero-dimensional (0D) spherical micro-sized aluminum oxide (Al_2_O_3_) on the thermal conductivity of epoxy resin was investigated. With 80 wt.% hybrid Al_2_O_3_–BN filler contents, the thermal conductivity of the EP composite reached 1.72 W·m^−1^·K^−1^, increasing approximately 7.8-fold with respect to the pure epoxy matrix. Furthermore, different important properties for the application were analyzed, such as Fourier-transform infrared (FTIR) spectra, viscosity, morphology, coefficient of thermal expansion (CTE), glass transition temperature (T_g_), decomposition temperature (T_d_), dielectric properties, and thermal infrared images. The obtained thermal performance is suitable for specific electronic applications such as flip-chip underfill packaging.

## 1. Introduction

Over time, the different needs of customers, whether domestic or work-related, have led to the constant advancement in electronics applications; one example is the pursuit of a continual increase in processing speed, which implies an increase in the connection density [1]. This constant development has resulted in the miniaturization of transistors, enabling more transistors to be attached and combined into a single device, leading to superior performance. This also implies changing the dimension of the devices to be as small as possible, which has led the semiconductor industry to explore materials on a smaller scale, known as micro-electronic technology [2]. With the rapid growth of this technology, the electronic components have progressively achieved increasing levels of processing speed [3]. Nevertheless, these improvements have also resulted in an increase in heat flux from electronic devices, owing to the large amount of heat generated during the operation of electronic devices [4,5,6]. Numerous studies and experience have demonstrated that the stability of electronic devices is directly affected by a rise in operating temperature owing to heat accumulation, which also reduces the lifespan of the device [7]. Therefore, it is critical for the heat produced from the devices to be dissipated as quickly and effectively as possible to maintain the working temperatures of the device at an acceptable level. Recently, the realization of heat dissipation has become the focus of electronic encapsulation and the study of underfill, which is one of the most recent techniques in this area. In the future, an ideal underfill should not only possess high thermal conductivity, but also a low coefficient of thermal expansion (CTE) because underfilling enhances the connection strength of electrical contacts and compensates for differences in the thermal expansion rates of two joining materials, which could lead to product failure [8].

With the rapid advances in material science and technology, numerous new materials such as carbon nanotubes (CNTs), carbon fibers, and graphene sheets have been used to enhance the thermal conductivity of the encapsulants [9,10]. Nonetheless, there were also some aspects to consider such as mass production and the high cost, limiting the extensive use of such new materials in electronic encapsulation [11]. The majority of underfill materials are two-phase compounds, epoxy matrices with thermally conductive but electrically insulating fillers, for example, an epoxy matrix packed with silica particles. Even if the epoxy with silica particles exhibited excellent performance, optimizations of the mechanical strength, thermal stability, and higher thermal conductivity of the underfill are regularly demanded for the flip-chip junctions of the forthcoming generation [12]. The inclusion of high-thermal-conductivity fillers has been extensively utilized to upgrade the thermal conductivity of the polymer matrix, which is usually very low (0.1–0.5 W·m^−1^·K^−1^) [13,14]. Typical filler materials consist of carbon-based materials (e.g., graphene, diamond, and CNT), metals (e.g., Cu, Ag, and Al), and ceramics (e.g., boron nitride and alumina nitride). To obtain high thermal conductivity for the underfill application, a high filler loading (>50 wt.%) was required for traditional micro-sized flake-like or spherical fillers. Such large loading mostly resulted in poor processability/flowability and elevated cost, restricting practical applications for IC encapsulation [15,16]. Alumina is a promising filler with high thermal conductivity (~30 W·m^−1^·K^−1^), but without electrical conduction and with a spherical-like particle shape [17], which makes it good for easy processability and flowability, implying that it is possible to achieve very high filler loading (>70 wt.%). Comparably, boron nitride (BN) possesses notably high thermal conductivity in the plane (220–420 W·m^−1^·K^−1^) [18], and such properties for underfill applications in the future will have a particularly high demand. However, several studies found that it is challenging to improve the dispersibility and achieve a high filler ratio for BN particles to obtain such thermal conductivity, particularly when using small particle sizes (0.01–1 μm), owing to the nature of flake-like particles [7,13,19]. Thermal conductivity is highly dependent on the networks created by the fillers in terms of heat pathways. Different factors affect the formation of these conductive channels, such as the filler content, filler distribution, the intrinsic characteristics of the filler (e. g., size, shape, and type), surface modification, and polymer matrix–filler interface interaction [7,12,14,20,21]. To enhance the use of fillers, the formation of continual heat pathways in the matrix by synergizing the properties of different materials and creating a 3D thermal conductive structure is a beneficial way to decrease the thermal resistance of the filler–matrix interface, thus increasing the thermal conductivity. Some authors reported superior thermal conductivities for composites using hybrid fillers with different dimensions. Fang et al. [22] found that both filler structure and surface modification play important roles in thermal conductivity. Zhang et al. [23] concluded that the addition of nanodiamond particles between BN nanosheet layers prevents agglomeration and improves mechanical and thermal properties. Pan et al. [24] reported that the AIN/hBN hybrid composite has thermal conductivity of 1.04 W·m^−1^·K^−1^, 3.8 times that of neat PTFE (0.272 W·m^−1^·K^−1^), and also exhibits higher thermal conductivity than both AIN and hBN single fillers. The above discussion highlights the enormous benefit of using hybrid fillers, which boost the possibility of creating more effective thermal links between the fillers and the polymer interface, creating a three-dimensional (3D) thermal conductive structure, thereby increasing the thermal conductivity. However, thermal resistance between the filler and matrix interface will exist owing to the difference in structure. Consequently, polymer composites with higher thermal conductivity can be achieved by decreasing the interfacial thermal resistance. The surface modification of fillers has resulted in an enhancement of filler interaction and polymer matrix [25,26]. Moreover, the underfill material must possess a high electrical resistivity, which is necessary to prevent damage or interference in the electrical circuit between the chip and the board, as well as a low dielectric constant to ensure a fast transmission speed of signals; from the literature, it can be found that these properties depend mainly on the nature of the filler and the frequency. Huang et al. [27] showed the dependence of the dielectric constants of the epoxy composites filled with BN on the frequency and the intrinsic characteristics of fillers.

In this study, a simple procedure was carried out to facilitate the productivity of possible IC underfill encapsulants in the near future. The various composite viscosities served as a key point for identifying the optimal bimodal distribution of the hybrid filler composed of two-dimensional (2D) flake-like BN and zero-dimensional (0D) spherical micro-sized Al_2_O_3_ particles. The impact of the hybrid filler on the enhancement of the epoxy resin was investigated by analyzing different properties, such as thermal conductivity, infrared thermal imaging, coefficient of thermal expansion, glass transition temperature, decomposition temperature, and electrical properties.

## 2. Experimental

### 2.1. Materials

Bisphenol-F epoxy (830LVP) was obtained from the DIC Company, Tokyo, Japan. The hardener was an Amine type (KAYAHARD-AA) acquired from Nippon Kayaku Company, Tokyo, Japan. The silane (Xiameter OFS-6040) was purchased from Dow Chemical Company, Midland, MI, USA. Boron nitride flakes with purity (99%) were purchased from King Meitek Industrial Co., Ltd. (Kaohsiung, Taiwan). High-purity (99.98%) spherical micro-sized alumina and alumina nanoparticles were purchased from Showa Denko Company, Tokyo, Japan. Detailed information on the matrix and filler are listed in Table 1.

### 2.2. Epoxy Resin System Selection

The epoxy binder system was composed of the epoxy resin, hardener, and silane. Foremost, this study investigated different types of epoxy binder systems by combining different types of the epoxy resin, hardener, and silane. First, the epoxy and the hardener were blended with a weight ratio of 1:0.8 in a Planetary Centrifugal Mixer (THINKY mixer) at 2000 rpm for 20 min. After that, the obtained mixture was mingled with the silane in the same centrifugal mixer with a proportion of 1 wt% of the previous solution. Subsequently, the viscosity of each epoxy binder system was measured. Finally, one epoxy binder system was selected on the basis of the lowest viscosity measured. This system is comprised of bisphenol-F epoxy (830LVP), hardener (KAYAHARD-AA), and silane (Xiameter OFS-6040), denoted by (EP).

### 2.3. Fabrication of m-Al_2_O_3_-BN/EP Composites

**Figure 1:**
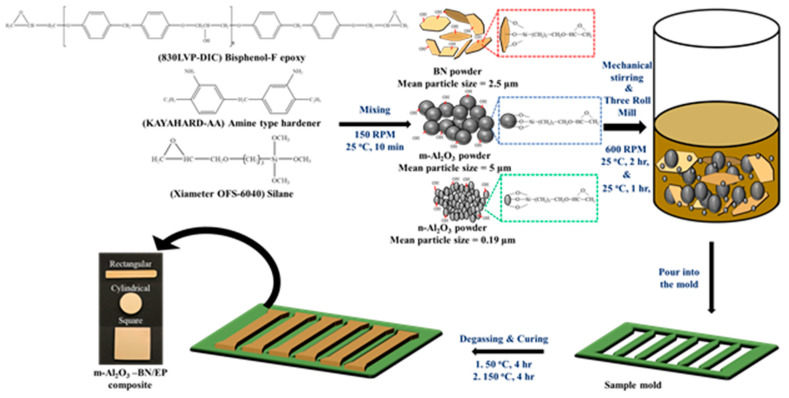
Schematic diagram of EP composites fabrication.

**Figure 2:**
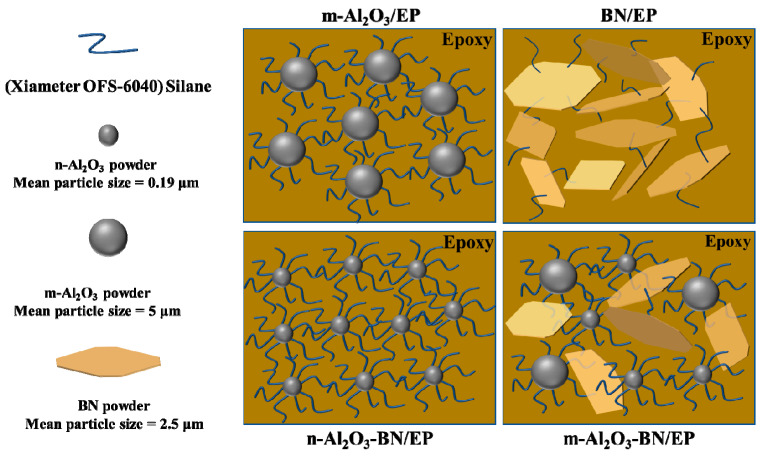
Schematic of the mechanism of surface modification for Al_2_O_3_ and BN fillers with the epoxy binder system.

The fabrication schematic of the Al_2_O_3_–BN/EP hybrid composite is illustrated in Figure 1, and Figure 2 shows the mechanism diagram for all the EP composites. First, only the pure epoxy was preheated at 90 °C for 6–8 h to melt the crystals formed at 25 °C, and all the fillers were preheated at 100 °C for 10–12 h to ensure that the water moisture was removed. Subsequently, the fillers and NaOH were mixed for 24 h at 120 °C and then washed several times with deionized water; next, the solution was filtered and finally placed into the oven for drying for 24 h at 80 °C. Next, 830LVP and KAYAHARD-AA were mixed at 150 rpm for 10 min at 25 °C with a weight ratio of 1:0.8, after which the Xiameter OFS-6040 was mixed with the proportion of 1 wt.% of the previous solution. Afterward, the Al_2_O_3_ and BN fillers were added to the preceding mixture at different weight percentages (wt.%), followed by hand mixing for 10–15 min. Subsequently, the mixture was blended by mechanical stirring at 600 rpm for 2 h at 25 °C; later, the dispersion of the filler into the epoxy matrix was made using a three-roll miller for 1–1.5 h. Next, it was mechanically stirred one more time at 600 rpm for 30 min at 25 °C to create a homogeneous mixture. Thereafter, the final solution was gently placed into a Teflon sample mold to perform different tests. The sample mold was then set into a vacuum chamber for the degassing process by utilizing an oil pump for 2–4 h at 50–110 °C depending on the viscosity of the system. Simultaneously, the viscosity of the final solution was measured. Finally, the sample was cured for 4 h at 150 °C, and m-Al_2_O_3_–BN/EP was acquired. Similarly, the remaining EP composites with or without fillers were also prepared using the same procedures, denoted by EP (without filler), m-Al_2_O_3_/EP (with spherical micro-sized Al_2_O_3_ filler), n-Al_2_O_3_/EP (with Al_2_O_3_ nanoparticle filler), BN/EP (with BN filler), and m-Al_2_O_3_-BN/EP (with m-Al_2_O_3_ and BN hybrid filler).

### 2.4. Characterization

The samples of a scanning electron microscope (SEM) were obtained by breaking the rectangular sample with pliers and a wire cutter. After that, platinum was coated on the broken surfaces and the morphology of the composites was observed using a field emission SEM (JSM-6500F). Fourier transform infrared spectroscopy (FTIR) was conducted using an FTIR spectrometer (Jasco FT/IR-4600). The samples were placed on a thin KBr plate and then scanned under infrared light from 4000 to 600 cm^−1^. The viscosity of the samples was estimated using a modular compact rheometer (MCR 102 Anton Paar), and the measurement was performed immediately after the second mixing of the processing at 25 °C and a constant shear rate of 10 (1/s). The thermal conductivity and thermal diffusivity of the samples were measured using a thermal constant analyzer (Hot Disk TPS 2500 S). The samples used were cylindrical in shape, as described above. The composite infrared thermal images were obtained using a thermographic camera (FLIR ONE Pro-iOS) under a room temperature range of 25–30 °C. The linear coefficient of thermal expansion (CTE) was determined using a thermomechanical analyzer (TMA Q400 TA instruments). The samples were placed under a nitrogen atmosphere and heated at a ramping rate of 5 °C/min to 120 °C. The TGA of the composites was characterized by TA instruments Q500 under an N_2_ atmosphere within the temperature range of 30–700 °C at a ramping rate of 10 °C/min. The composites dielectric properties were acquired by Agilent 8722ES, the samples utilized were square shaped, as described previously, the measurements were conducted under a room temperature range of 23–25 °C. An electrode sensor (85052B) was used and the applied voltage is based on the built-in voltage of the precision impedance analyzer.

## 3. Results and Discussion

### 3.1. Underfill Technique

**Figure 3:**
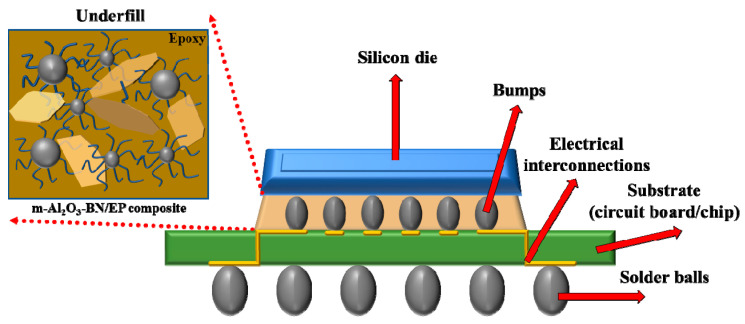
Schematic of the flip-chip underfill packaging.

Of all the interconnects, the flip-chip has attracted considerable attention because of its high number of I/Os, short length, and low inductance. However, the lower contact area between the substrate and chip also causes some reliability problems, such as thermal performance and mechanical reliability owing to thermal expansion differences among them. One of the current techniques to assist in the heat dissipation of the IC is underfill encapsulation. Figure 3 represents a schematic drawing of the solder-bumped flip-chip and the underfill on a circuit board. The underfill is usually distributed along the area of the chip and stream into the gap between the chip and the substrate by capillary force, which fills the gap between the substrate and the chip, providing an effective heat path. Thus, this reduces the interstitial air gap trapped as a result of inaccurate joining of the surfaces, which significantly decreases the capacity to dissipate heat owing to the low thermal conductivity of the air (0.026 W·m^−1^·K^−1^). First, the purpose of the underfill is to protect the bumps of the chip from mechanical stress on its substrate or another chip. Consequently, a low CTE is required. Moreover, dissipation of the heat is generated in the chip by conducting heat to the substrate; hence, high thermal conductivity is essential [2,8]. Lastly, the procedure is time-consuming and costly. Therefore, the viscosity of the underfill is a decisive factor in decreasing the processing time.

### 3.2. FTIR

**Figure 4:**
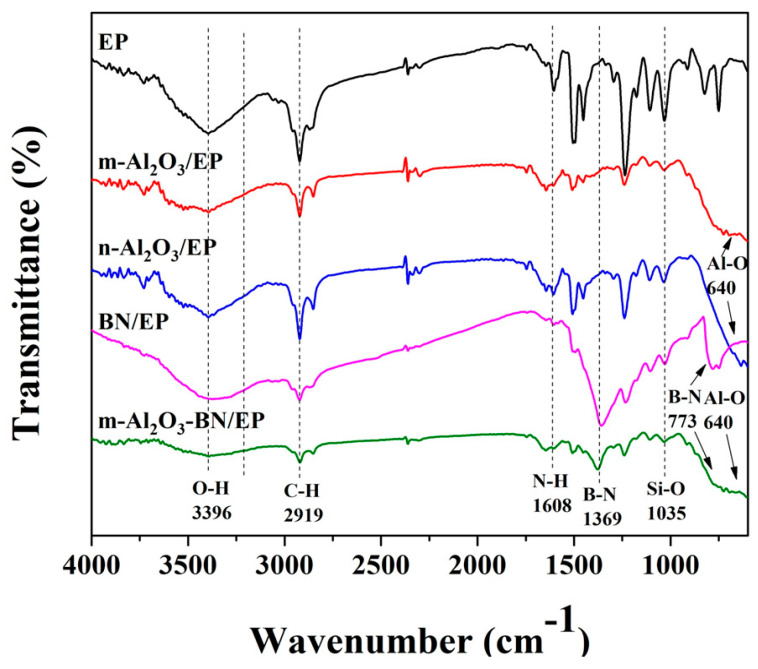
FTIR spectra of different EP composites.

Figure 4 illustrates the FTIR spectra chart of the EP and the various composites at maximum filler weight percent possibly achieved in this study. Two strong peaks at 773 cm^−1^ and 1369 cm^−1^ were observed in the BN/EP composite, corresponding to the bending vibration and stretching vibration of the B-N bond, respectively [28,29]. This peak also appeared in the m-Al_2_O_3_-BN/EP composite. The peaks at 1035 cm^−1^ designated as Si-O vibration [30,31,32], showed in all EP composites and in the pure epoxy. In the epoxy, the peak is very sharp, but with the addition of filler content, the peak shrinks owing to a decrease in the silane volume in the total composite solution. Meanwhile, in addition to the Al-O bond appearing at 640 cm^−1^ [33,34] on m-Al_2_O_3_-/EP, a similar peak behavior was also observed for the n-Al_2_O_3_/EP and m-Al_2_O_3_-BN/EP composites. Furthermore, the broad peak absorption at 3396 cm^−1^ corresponds to the stretching vibration of –OH [28,35], which may be attributed to the –OH on the silane and the water molecules that may be absorbed from the environment on the filler surface. All of these results show evidence of the presence of both Al_2_O_3_ and BN fillers as well as the silane in different fabricated samples.

### 3.3. Rheological Study

**Figure 5:**
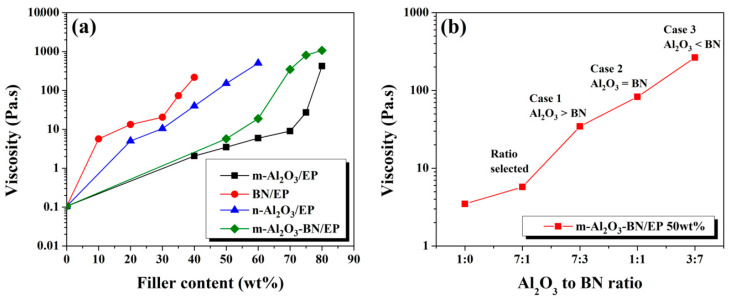
Viscosity charts of (**a**) the distinct EP composites as a function of filler content and (**b**) m-Al_2_O_3_-BN/EP with 50 wt% as a function of Al_2_O_3_ to BN ratio.

As explained in Section 2.2, this research experiment was mainly based on experimentally finding the hybrid filler optimal filler distribution composed of m-Al_2_O_3_ and BN fillers, primarily by observing the rheological behavior of each different composite with single fillers. The viscosities of the different EP composites are shown in Figure 5a, which displays the viscosity variation of different composites as a function of filler content. The viscosity of the pure epoxy obtained was 0.105 Pa·s. In addition, the viscosities obtained for the different EP composites at maximum filler loading were as follows: m-Al_2_O_3_/EP 80 wt.% (424 Pa·s), n-Al_2_O_3_/EP 60 wt.% (503 Pa·s), BN/EP 40 wt.% (217 Pa·s), and m-Al_2_O_3_–BN/EP 80 wt.% (1069 Pa·s). It was observed from the results that the viscosity is influenced by the particle size, particle shape, and amount of filler content [15,36,37,38,39]. The BN composites started exhibiting a prompt increase in viscosity at low filler content, which was related to their flake-like particle shape. Similarly, the n-Al_2_O_3_/EP composites exhibited the same behavior owing to the high surface area of Al_2_O_3_ nanoparticles; however, the m-Al_2_O_3_/EP composites exhibited much lower viscosity throughout the entire range of filler loading, presumably owing to the spherical shape and larger particle size, allowing easy flow pathways for the polymer. Furthermore, the viscosities of the m-Al_2_O_3_-BN/EP composites were higher than those of the m-Al_2_O_3_/EP composites throughout the entire range of filler content, which was ascribed to the addition of BN filler into the compound, thus generating a stronger interfacial interaction between the filler and the epoxy matrix. Figure 5b presents the viscosity variation of m-Al_2_O_3_-BN/EP with 50 wt.% as a function of the Al_2_O_3_–BN ratio. According to the results of the viscosities obtained for m-Al_2_O_3_/EP, a filler content of 50 wt.% was selected because, after this loading, the viscosity started showing a significant increase in value, as illustrated in Figure 5a; thus, this point was ascribed as the percolation threshold for that composite. Additionally, the spherical shape of m-Al_2_O_3_ allowed it to achieve a much higher filler loading than when using n-Al_2_O_3_, thereby increasing the potential to achieve a higher packing density in the epoxy matrix, resulting in an increase in thermal conductivity. Three cases were proposed, as shown in Figure 5b: Case 1 (Al_2_O_3_ wt.% > BN wt.%), Case 2 (Al_2_O_3_ wt.% = BN wt.%), and Case 3 (Al_2_O_3_ wt.% < BN wt.%). It can be noted that, with a further increase in BN filler content, the viscosity of the bimodal distribution compound with m-Al_2_O_3_/EP and BN/EP increased considerably; therefore, a ratio between Case 1 and the origin (m-Al_2_O_3_/EP 50 wt.% without BN) was selected because the region of the curve possessed the ratios that led to the lowest viscosities of this particular hybrid composite. The selected ratio was 7:1, and this ratio was used for fabricating the m-Al_2_O_3_–BN/EP at different filler loadings. In addition, a similar graph is presented in different sections of the paper for further information on this composite system.

### 3.4. Thermal Conductivity of EP Composites

**Figure 6:**
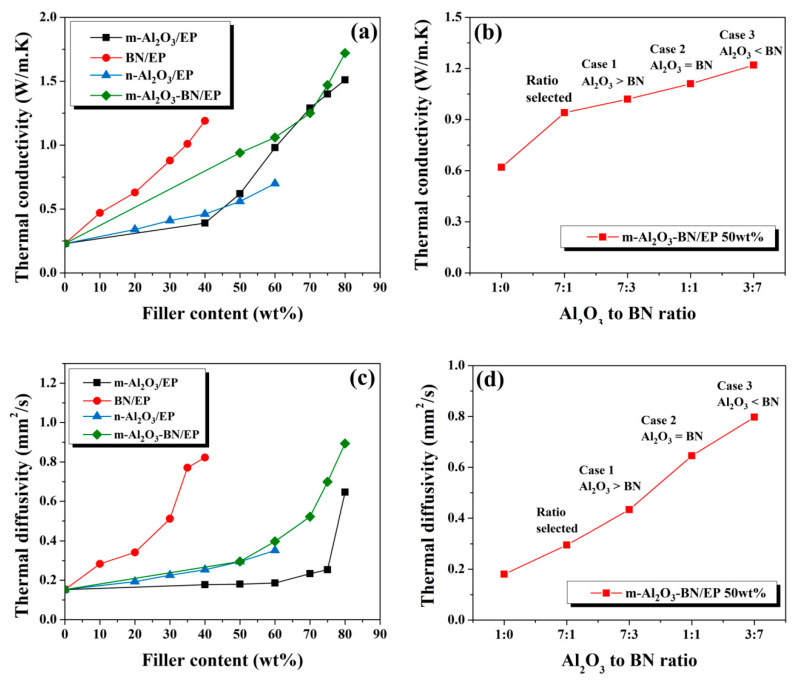
Thermal conductivity of (**a**) the distinct EP composites as a function of filler content and (**b**) m-Al_2_O_3_-BN/EP with 50 wt% as a function of Al_2_O_3_ to BN ratio. Thermal diffusivity of (**c**) the distinct EP composites as a function of filler content, and (**d**) m-Al_2_O_3_-BN/EP with 50 wt% as a function of Al_2_O_3_ to BN ratio.

The thermal conductivity of each sample was measured five times, and the average value was chosen as the representative value (Figure 6). The thermal conductivities and diffusivities of the different EP composites are shown in Figure 6a,c, respectively. Figure 6a displays the thermal conductivity variations of the different composites as a function of filler content. In general, for all cases, the thermal conductivity increased with filler loading, and it was noted that the thermal conductivity is affected by the particle size and shape of filler particles [1,7,15,19,36,37,40,41,42,43,44]. The epoxy binder system exhibited a very low thermal conductivity of 0.22 W·m^−1^·K^−1^. The thermal conductivity of the m-Al_2_O_3_/EP composites was much higher than that of the n-Al_2_O_3_/EP composites. For example, the thermal conductivity of m-Al_2_O_3_/EP reached 0.98 W·m^−1^·K^−1^, whereas that of n-Al_2_O_3_/EP was 0.70 W·m^−1^·K^−1^ for the same filler loading of 60 wt.%. This may be attributed to the spherical-like particles of m-Al_2_O_3_/EP that possess smaller contact areas between particles; hence, they can be dispersed better into the epoxy matrix. Meanwhile, the alumina nanoparticles of n-Al_2_O_3_/EP have a very small particle size and exhibit irregular shape, thereby tending to form more aggregation groups, resulting in a more difficult particle dispersion owing to the higher surface area. Similarly, the thermal conductivity of the BN/EP composites was 1.19 W·m^−1^·K^−1^ for 40 wt.% filler content, which is attributed to the very high intrinsic thermal conductivity of BN particles. The highest thermal conductivity of 1.72 W·m^−1^·K^−1^ was achieved for the m-Al_2_O_3_–BN/EP with 80 wt.%, increasing approximately 7.8-fold with respect to the pristine epoxy matrix and approximately 15% more than that of the m-Al_2_O_3_/EP 80 wt.% (1.51 W·m^−1^·K^−1^), which can be ascribed to the addition of a small amount of BN filler, together creating a 3D thermal conductive structure with a better formation of heat pathways. Figure 6b presents the thermal conductivity variation of m-Al_2_O_3_–BN/EP with 50 wt.% as a function of Al_2_O_3_–BN ratio. The results acquired indicate that, with a further increase in BN filler content, the thermal conductivity of the m-Al_2_O_3_–BN/EP also increases significantly; however, increasing the BN content may increase the thermal conductivity at this filler content, but at the cost of an exponential increase in the viscosity of the system, as observed from the results in Figure 5b. Accordingly, this prevents achieving a higher packing density in the epoxy matrix, thereby limiting the addition of filler content for this composite and finally restricting the capability of obtaining a higher thermal conductivity.

### 3.5. Composites Thermal Management Capacity

**Figure 7:**
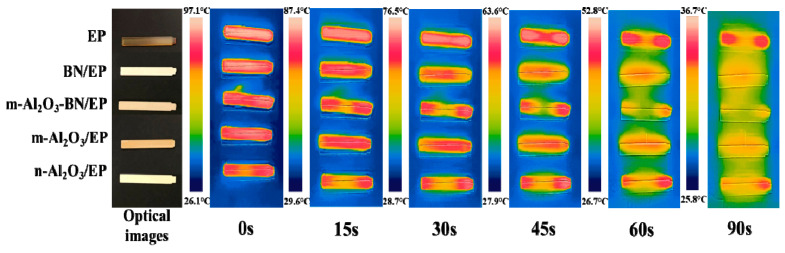
Optical and infrared thermal images of EP composites.

Figure 7 and Video S1 (in the Supplementary Materials) show the optical images of five kinds of EP and different composites with rectangular shape after cutting and polishing. The pure epoxy resin showed a yellow color, the BN/EP presented a white color, the m-Al_2_O_3_–BN/EP exhibited a soft beige color, the m-Al_2_O_3_/EP possessed a dark beige color, and the n-Al_2_O_3_/EP showed a pale yellow color. Additionally, three sample shapes were made for the different tests presented in this research, described as follows: rectangular shape (88 mm in length, 12 mm in width, and ~3 mm thickness); cylindrical shape (35 mm in diameter and 8 mm in height); square shape (55 mm in length, 55 mm in width, and ~3 mm thickness). To estimate the heat dissipation ability of the hybrid composites, the temperature–time variations of diverse samples during cooling were carried out. Each sample was placed at 100 °C for 5 h to ensure that the experimental conditions were equal [43,45,46,47]. As illustrated in Figure 7, the single filler composites exhibited quicker heat dissipation ability in comparison with EP. Furthermore, the m-Al_2_O_3_–BN/EP composite showed a superior heat-transfer potential, attributed to its higher thermal conductivity and, therefore, it derived a more rapid thermal response. For instance, after cooling for 90 s, the temperature of the Al_2_O_3_–BN/EP composite reached approximately 26.5 °C, which is notably lower than that of the pure epoxy resin (36.7 °C). As a result of the high thermal conductivity exhibited, the hybrid filler composites possess superior heat dissipation capability, making them potentially useful as an underfill encapsulation material for electronic packaging.

### 3.6. Glass Transition Temperature and Coefficient of Thermal Expansion of EP Composites

**Figure 8:**
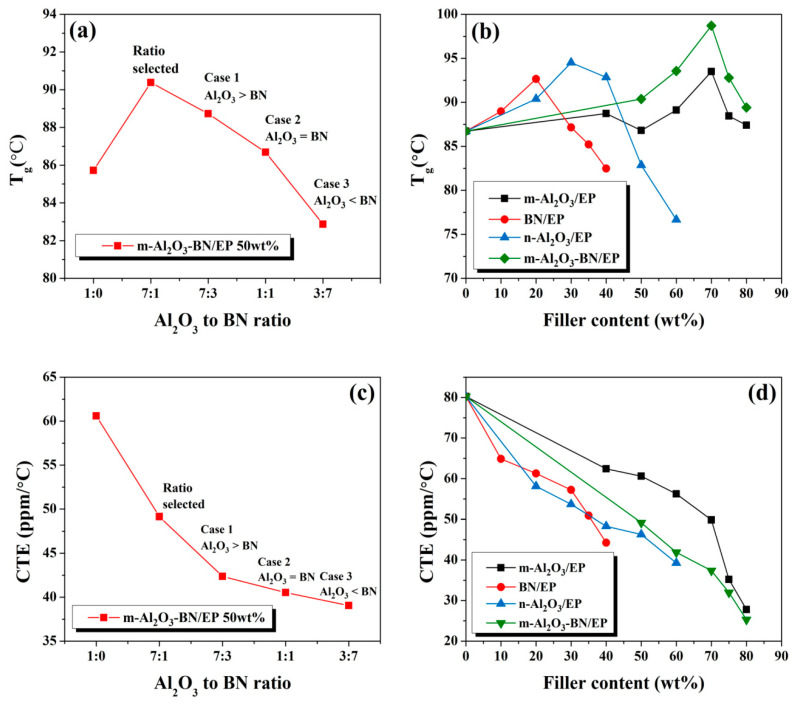
Glass transition temperature (T_g_) of (**a**) m-Al_2_O_3_-BN/EP with 50 wt% as a function of Al_2_O_3_ to BN ratio and (**b**) the distinct EP composites as a function of filler content. Coefficient of thermal expansion (CTE) below T_g_ of (**c**) m-Al_2_O_3_-BN/EP with 50 wt% as a function of Al_2_O_3_ to BN ratio and (**d**) the distinct EP composites as a function of filler content.

The glass transition temperatures (T_g_) of the EP composites are illustrated in Figure 8a,b. Figure 8a presents the T_g_ variation of m-Al_2_O_3_–BN/EP with 50 wt.% as a function of the Al_2_O_3_–BN ratio. The results indicate that the ratio distribution in the hybrid filler plays an important role in the value of T_g_. For this particular bimodal ratio distribution, as long as more BN filler content is added, the T_g_ tends first to increase at low wt.% content and then, at high wt.% content, decreases significantly, reaching below that of pure epoxy (86.72 °C). Figure 8b displays the T_g_ values of the different EP composites as a function of wt.%. The chart shows that the composite T_g_ is influenced by the particle size and amount of filler content [12,36]. The addition of n-Al_2_O_3_ and BN fillers in the epoxy initially increased the T_g_ slightly at low wt.% and decreased it considerably at higher wt.% below that of EP, reaching a T_g_ of 76.65 °C and 82.47 °C, respectively. It was found in different studies [48,49,50] that high surface area fillers exhibit more interaction with the epoxy matrix, which may cause alterations in crosslinking density, leading to a lower T_g_. Moreover, the m-Al_2_O_3_ and m-Al_2_O_3_–BN/EP fillers exhibited similar behavior as the previous fillers. The increase in T_g_ suggests that the interaction between the epoxy matrix and micro-sized filler can successfully restrict the epoxy chain motion, and this interaction improves with higher filler content and optimal ratio distribution [19,40]. However, at high wt.%, they maintained similar values of T_g_ compared with that of EP, reaching a T_g_ of 87.39 °C and 89.41 °C, respectively. Furthermore, the T_g_ exhibited a decreased trend for m-Al_2_O_3_ and m-Al_2_O_3_-BN/EP after 70 wt.% of filler content, which may be ascribed to an increase in viscosity. The removal of interstitial air trapped at the epoxy–filler interface becomes complicated with an increase in viscosity; consequently, there is a poor interaction between the epoxy matrix and the filler, thus inhibiting effective limitation of the epoxy chain motion. The CTE of the epoxy matrix will considerably improve upon adding any of the fillers because the intrinsic CTE values of Al_2_O_3_ and BN are much lower than those of epoxy (80.25 ppm/°C), as observed in Figure 8c,d and also verified from the literature [12,36]. The minimum value of CTE was obtained from m-Al_2_O_3_–BN/EP at 80 wt.%, achieving 25.28 ppm/°C, which is a desirable value for underfill applications. It was observed that the CTE of composites not only relied on the filler content, but also on the filler size. For example, the CTE of the n-Al_2_O_3_ composite with 40 wt.% was smaller than that with m-Al_2_O_3_ at the same loading level. This was attributed to the increased surface area of the alumina nanoparticles, which presented a greater interfacial region between the matrix and the filler, thereby restraining the expansion of the epoxy matrix. Comparably, the CTE of the m-Al_2_O_3_ and m-Al_2_O_3_-BN/EP composites at very high filler loadings (>70 wt.%) decreased enormously, which may be attributed to two reasons. First, at very high loading, the viscosity of the system exponentially increases, thus providing significant physical and mechanical constraints to the epoxy matrix. Second, the synergistic effect of the hybrid filler may generate an effective huge interfacial interaction between the matrix and the filler, thus limiting the expansion of the epoxy [48,49,50].

### 3.7. Morphological Observation of m-Al_2_O_3_-BN/EP Composites

**Figure 9:**
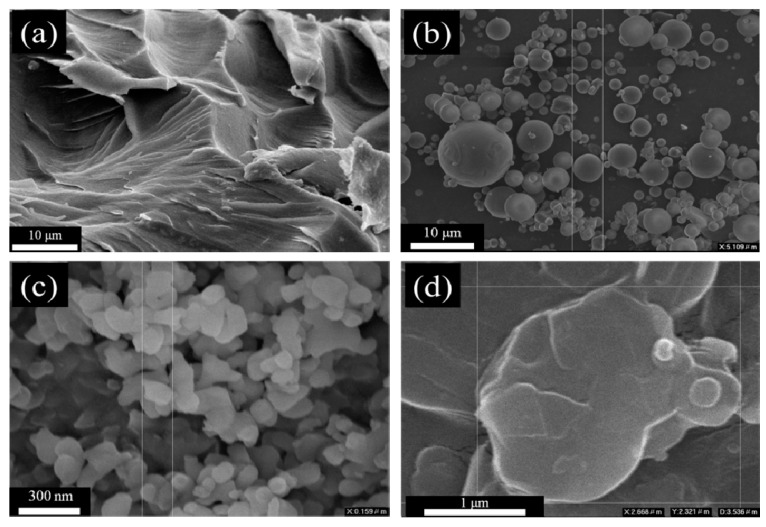
SEM micrographs of (**a**) EP; (**b**) m-Al_2_O_3_; (**c**) n-Al_2_O_3_, and (**d**) BN.

**Figure 10:**
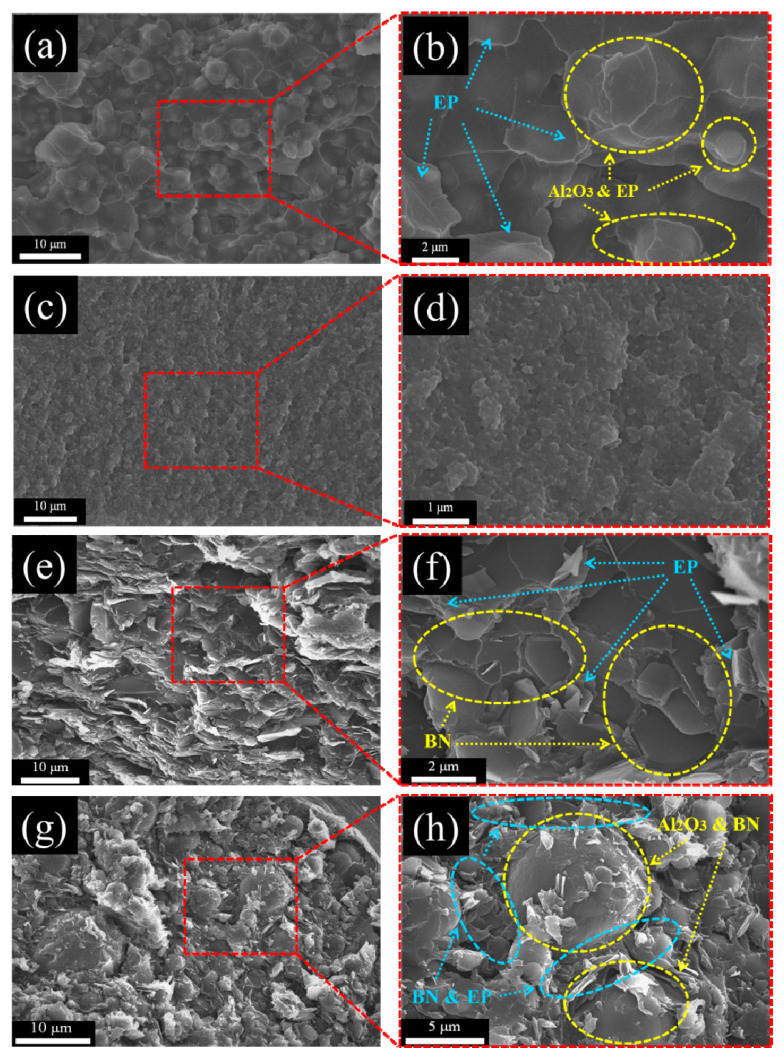
SEM images of EP composites with (**a,b**) m-Al_2_O_3_ 80 wt%; (**c**,**d**) n-Al_2_O_3_ 60 wt%; (**e**,**f**) BN 40 wt% and (**g**,**h**) m-Al_2_O_3_-BN 80 wt%.

The cross-section of the EP and the morphology of the different fillers are displayed in Figure 9. The cross-sectional morphologies of m-Al_2_O_3_/EP, n-Al_2_O_3_/EP, BN/EP, and m-Al_2_O_3_–BN/EP using SEM are presented in Figure 10. As shown in Figure 10a,b, the m-Al_2_O_3_ particles are well dispersed in the epoxy matrix, which can be ascribed to the spherical shape of the m-Al_2_O_3_ particles. However, it is also known that the spherical particles have less contact area; therefore, even with a very high filler loading (80 wt.%), they do not form effective thermal conductive pathways. Figure 10c,d show that the n-Al_2_O_3_ nanoparticles are dispersed with some aggregation, which is very typical because a smaller particle leads to a higher specific surface area, increasing the intermolecular forces and facilitating the formation of more aggregation, thereby hindering the dispersion of the filler particles into the epoxy matrix. As displayed in Figure 10e,f, the BN particles seem to be dispersed and have some heat pathways, but with some aggregation; this can be attributed to the flake-like particles, which have a larger surface area, thus facilitating the mutual attachment of the particles. This means that a flawless thermally conductive pathway is difficult to achieve with a single filler because of the presence of a thick epoxy matrix layer between the fillers. However, continual linkage with slight aggregation can be observed in the m-Al_2_O_3_–BN/EP composite, as shown in Figure 10g,h, which indicates that the hybrid fillers feature a more functional interfacial connection compared with the single fillers. The 2D flake-like BN has a smaller particle size than the 0D spherical micro-sized Al_2_O_3_; therefore, the BN particles with the assistance of the shear motion during the stirring process can easily enter the gap of the micro-sized Al_2_O_3_, thereby avoiding a high level of agglomeration to form thermal links among the BN and creating a 3D thermal conductive structure. Hence, more effective thermal channels are formed in m-Al_2_O_3_–BN/EP composites.

### 3.8. Thermal Stability

**Figure 11:**
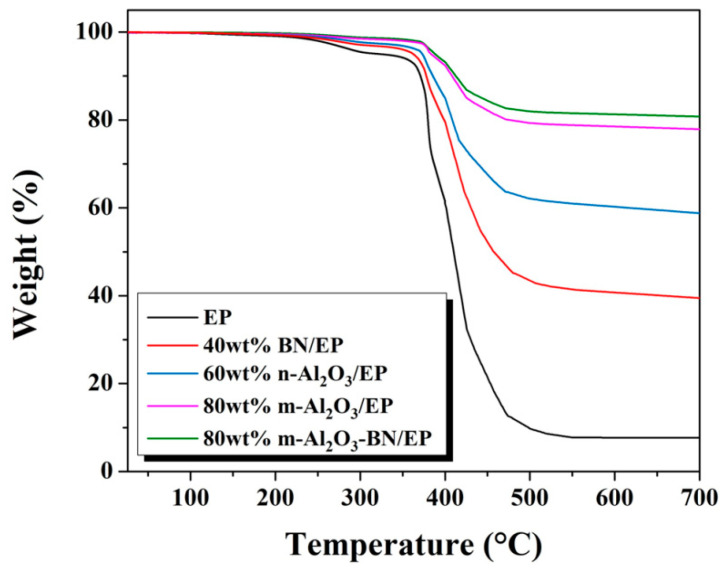
TGA curves of various EP composites.

Figure 11 illustrates the TGA curve of the EP and various composites at the maximum filler weight percentage possibly achieved in this research. The pure epoxy resin exhibits a slight weight loss starting at approximately 248.7 °C, and the sudden drop at approximately 350.2 °C corresponds to the thermal decomposition of EP, whereas the corresponding temperature of the single filler composites is significantly enhanced. A decomposition temperature of approximately 375 °C was acquired in the m-Al_2_O_3_–BN/EP composite. Moreover, the residual weight of the m-Al_2_O_3_–BN/EP composite reached 81%, which is an evident enhancement compared to 78% for m-Al_2_O_3_/EP, 59% for n-Al_2_O_3_/EP, 39% for BN/EP, and 8% for EP. The enhancement in thermal performance may be attributed to the combined effects of the hybrid fillers. The good dispersion of fillers in the hybrid composite boosts the interaction within the matrix and fillers, which can successfully restrict the kinetic motion of molecular chains from the matrix. Hence, this indirectly enhances the thermal stability of the composite materials. The outstanding thermal resistance of m-Al_2_O_3_–BN/EP composites can effectively guard the bumps and chip packaging against heat decomposition, which indicates considerable potential as an underfill encapsulation material for electronic packaging.

### 3.9. Electrical and Dielectric Properties

**Figure 12:**
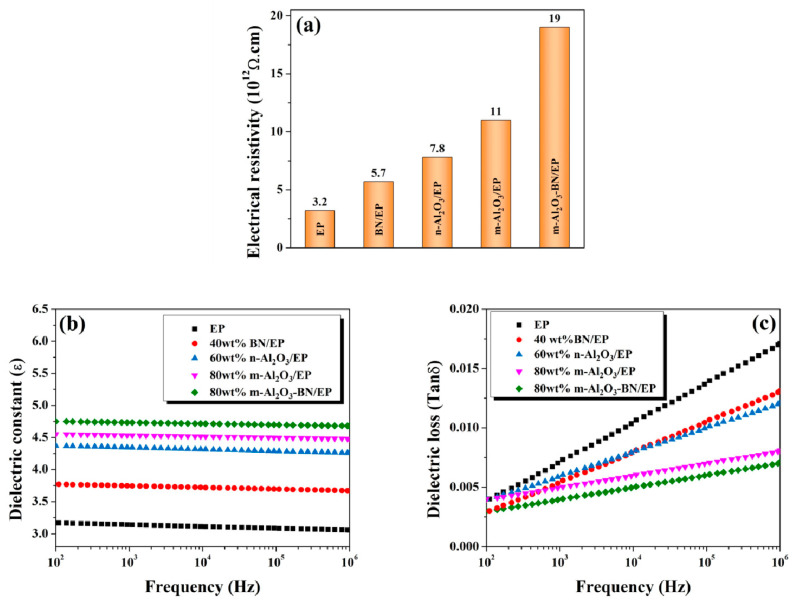
Electrical properties graphs of the different EP composites at maximum filler wt% achieved in this study: (**a**) Volume resistivity; (**b**) Dielectric constant versus frequency, and (**c**) Dielectric loss versus frequency.

To prevent damage or interference in the electric circuit between the chip and the board, the underfill material must ensure electrical insulation. Hence, the pure epoxy resin possesses an excellent insulating property, and the volume resistivity reached 3.2 × 10^12^ Ω·cm, as shown in Figure 12a. It can be observed that the composite resistivity increased after the filler content, which was attributed to the exceptional electrical insulation of Al_2_O_3_ and BN. The results obtained are much higher than the minimum value of a material to be considered an electrical insulator (10^9^ Ω·cm) [45,51,52]; thus, the fabricated EP composites are suitable for use in underfill encapsulation for IC. The dielectric constant (ε) symbolizes the capability of maintaining electric charges in an electrical field, whereby a low dielectric constant is favorable to obtain faster transmission speed of signals, which is desired for electronic encapsulation applications [53]. The ε values of the EP composites are illustrated in Figure 12b. Compared with the pristine epoxy, both single and hybrid EP composites exhibited slightly larger ε values. For example, ε increased from 3.17 for EP to 4.75 for m-Al_2_O_3_–BN/EP. An increase in ε may be ascribed to the intrinsic ε value of Al_2_O_3_ and BN fillers. It was also observed that the EP composites ε decreased very slightly with increasing frequency, which was attributed to the dipole and interfacial polarization also known as Maxwell–Wagner–Sillars (MWS) [54,55,56]; studies have found that a rise in MWS polarization will be generated at lower frequencies, leading to a field intensification in the epoxy matrix, thus increasing the polarization. Figure 12c shows the dielectric loss (tanδ) of the EP composites, which is related to the energy loss resulting from the heating generated during the alternation of the polarity of the dielectric material surfaces under an alternating electric field [57]. Compared with EP, both single and hybrid EP composites exhibited slightly lower tanδ values. Moreover, it was noted that the EP composites’ tanδ increased quite slightly with increasing frequency, which was ascribed to the mismatch between the variation of the alternating electric field and the relaxation time of the dipolar group polarization [39,40]. Even if the tanδ for all EP composites increased slightly at higher frequencies, it was still below 0.05, exhibiting their potential application in electronic packaging [57].

## 4. Conclusions

In this study, thermally conductive EP composites with 0D Al_2_O_3_ and 2D BN hybrid fillers were fabricated by mechanical stirring and degassing. Additionally, the hybrid filler optimal filler distribution of the mentioned composite was found experimentally by observing the rheological behavior of each composite with single fillers and selecting the hybrid filler ratio which approximately led to the lowest viscosity. The resulting m-Al_2_O_3_–BN/EP hybrid composites exhibited better formation of thermal transport pathways. Therefore, the thermal conductivity of EP was considerably enhanced. As long as the filler particle size used decreases, the viscosity increases considerably because the epoxy moves more freely with the filler particles, rather than using small filler particles, because the surface area for the latter particles is much higher than that of the former particles, subsequently limiting the achievement of a high packing density in the epoxy matrix. Similarly, the comparison of thermal conductivity results revealed that the thermal conductivity of big particles was much higher than that of small particles because the big particles can form heat pathways more easily than the small ones. In addition, the CTE and T_g_ of composites were concluded to be influenced mainly by the intrinsic nature and morphology of the fillers. On the other hand, excellent electrical properties were found for the underfill encapsulation application. Further investigation with more sophisticated equipment is highly recommended to improve the flowability of the m-Al_2_O_3_–BN/EP composite, which might even increase the thermal conductivity owing to better dispersion and less air gas trapped in the final sample.

## Figures and Tables

**Table 1:** Detailed information of the matrix and filler.

Material	Mean Particle Size (μm)	Thermal Conductivity (W m^−1^K^−1^)	Density (g/cm^3^)
BN	2.5	220	2.1
Micro Al_2_O_3_	5	29	3.89
Nano Al_2_O_3_	0.19	27	
Epoxy		0.22	1.17

## Supplementary Materials

The following are available online at https://www.mdpi.com/2073-4360/13/1/147/s1, Video S1. A video showing a demonstration of the infrared thermal images of EP composites is provided.
